# Intraosseous basivertebral nerve ablation: Pooled long-term outcomes from two prospective clinical trials

**DOI:** 10.1016/j.inpm.2023.100256

**Published:** 2023-06-10

**Authors:** Matthew Smuck, Eeric Truumees, Kevin Macadaeg, Ashwin M. Jaini, Susmita Chatterjee, Joshua Levin

**Affiliations:** a Department of Orthopaedic Surgery, Division of Physical Medicine & Rehabilitation, Stanford University, 430 Broadway Street, Pavilion C 4th F^lo^or, Redwood City, CA, 94063, USA; b Ascension Texas Spine & Scoliosis, 1004 W 32nd St Suite 200, TX, 78705, USA; c Indiana Spine Group, 13225 N Meridian St, Carmel, IN, 46032, USA

**Keywords:** Axial chronic low back pain, Anterior column low back pain, Basivertebral nerve, Basivertebral nerve ablation, Modic changes, Vertebrogenic pain

## Abstract

**Background:**

Vertebrogenic pain is an established source of anterior column chronic low back pain (CLBP) resulting from damaged vertebral endplates with pain signals transmitted by the basivertebral nerve (BVN). Type 1 or Type 2 Modic changes on magnetic resonance imaging (MRI) are objective biomarkers for vertebrogenic pain. Radiofrequency ablation of the BVN (BVNA) has demonstrated both efficacy and effectiveness for the treatment of vertebrogenic pain in two randomized trials. Here, we report 3-year aggregate results from two prospective studies of BVNA-treated patients.

**Methods:**

Pooled results at 3 years post-BVNA are reported for two studies with similar inclusion/exclusion criteria and outcomes measurements: 1) a prospective, open label, single-arm follow-up of the treatment arm of a randomized controlled trial (RCT) comparing BVNA to standard care (INTRACEPT Trial), and 2) a prospective, open label, single cohort long-term follow-up study of BVNA-treated patients. Paired datasets (baseline and 3-years) for mean changes in Oswestry disability index (ODI) and numeric pain scores (NPS) were analyzed using a two-sided *t*-test with a 0.05 level of significance.

**Results:**

There were 95/113 (84%) BVNA patients who completed a 3-year visit across 22 study sites. At baseline, 71% of patients reported back pain for ≥5 years, 28% were taking opioids, 34% had spinal injections in the prior 12 months, and 14% had prior low back surgery. Pain and functional improvements were significant at 3 years with a mean reduction in NPS of 4.3 points from 6.7 ​at baseline (95% CI 3.8, 4.8; p<0.0001) and a mean reduction in ODI of 31.2 points from 46.1 ​at baseline (95% CI 28.4, 34.0; p<0.0001). Responder rates, using minimal clinically important differences of ≥15-points for ODI and ≥50% reduction in NPS from baseline to three years, were 85.3% and 72.6%, respectively (combined response 69.5%), with 26.3% of patients reporting 100% pain relief at 3 years. There was a 74% reduction in the use of opioids and 84% reduction in the use of therapeutic spinal interventions from baseline to 3 years. There were no serious device or device-procedure related adverse events reported through three years.

**Conclusion:**

Intraosseous BVNA demonstrates statistically significant, clinically meaningful, and durable improvements in pain and function through 3 years in patients with primary vertebrogenic low back pain. BVNA-treated patients significantly reduced opioid use and interventions for low back pain.

## 1 Background

Historically, clinicians treating chronic low back pain (CLBP) were challenged with limited objective differentiators to identify pain sources. This promoted non-specific diagnoses and a variety of treatment approaches, all with poor effect sizes, in addition to over-treatment [[1], [2], [3]]. CLBP is a symptom for a heterogenous group of causative conditions. Subgroupings of CLBP, ideally based on objective biomarkers, are necessary for more targeted and effective treatments to emerge [4]. Fortunately, advances in the understanding of spine biochemistry, biomechanics, epidemiology, and pathophysiology now enable a more sophisticated approach to the diagnoses and treatment of some CLBP subgroups [4].

Subgrouping CLBP typically begins with a clinical assessment of the patient, the pain location (e.g., lateral or midline) and movements or postures that exacerbate pain to determine the likely anatomic region generating pain (e.g., anterior or posterior column). When clinically indicated, imaging and response to diagnostic tests are used to attempt to isolate the pain source to specific anatomic structures. One structural source of anterior column pain, in the presence of Modic changes, is vertebral endplate pain (vertebrogenic pain) [4,5]. Bogduk et al. described four features necessary for a structure to qualify as a distinct source of low back pain: it must be 1) innervated, 2) susceptible to painful disease or injuries, 3) capable of causing pain similar to that seen clinically, and 4) diagnosable using a test with known reliability and validity [6]. Vertebrogenic pain meets these criteria. Vertebral endplates are richly innervated by the basivertebral nerve (BVN) [[7], [8], [9]] with a higher density of pain receptors than the adjacent intervertebral discs [10]. When vertebral endplates are damaged, interaction between the tissues of the endplate and the adjacent disc produces chronic inflammation followed by BVN sensitization [11]. Inflammation and edema from damaged endplates are visible as Type 1 and/or Type 2 Modic changes on MRI, a reliable and highly specific binary biomarker of vertebrogenic CLBP [12]. Modic changes, an objective imaging biomarker, in combination with clinical findings of anterior column pain proved useful to identify patients with vertebral endplate pain in two level 1 clinical trials [13,14].

With the BVN serving as the primary nociceptive input to the vertebral endplates, destruction of this nerve through radiofrequency ablation provides an opportunity to eliminate or reduce vertebrogenic pain. The efficacy of basivertebral nerve ablation (BVNA) was demonstrated in a pivotal RCT compared to sham [13] with long-term benefits maintained at 24 months and 5 years [15,16]. Subsequently, BVNA effectiveness and safety were examined in two additional studies: 1) an open label RCT comparing BVNA to non-surgical standard care with results reported through 6 months and BVNA-arm results reported through 24 months [14,17,18], and 2) a prospective single-arm cohort study [19] with BVNA results reported at 12 months [20]. Here, we report the 3-year results of BVNA-treated participants aggregated from these two studies.

## 2 Methods

### 2.1 Study design

The current study analyzed aggregate data from two prospective clinical trials sponsored by Relievant Medsystems, Inc. (Minneapolis, MN, USA). The two trials were 1) a prospective, open-label, single-arm follow-up study of the original BVNA treatment arm from the INTRACEPT RCT [[16], [17], [18]], and 2) a CLBP prospective, open label, single-arm cohort study of BVNA-treated patients with a subsequent long-term follow up study [19,20]. Participants in both studies were enrolled between September 2017 and February 2019 ​at 24 pain medicine and spine centers in the United States. Inclusion/exclusion criteria, follow-up visit schedules (excluding a 2-year visit for the single-arm cohort study), study endpoints, and protocol requirements were similar for the two studies, allowing for the data to be pooled. Each study was registered on ClinicalTrials.gov [NCT03246061 (INTRACEPT)], NCT03266107 (CLBP Single-Arm through 12 months), and NCT05207813 (CLBP Single-Arm Long-Term Study)]. The studies were compliant with Health Insurance Portability and Accountability Act (HIPAA), Good Clinical Practices, and the Declaration of Helsinki, and were conducted under Institutional Review Board approval and participant informed consent.

### 2.2 Study population

All participants enrolled in the two original studies had refractory CLBP for a minimum of six months, not responding to non-surgical treatment, with Modic changes (Type 1 and/or Type 2 Modic changes from L3-S1) as the imaging biomarker for primary vertebrogenic pain. The primary inclusion and exclusion criteria were the same for both studies (Table 1). Enrollment criteria allowed for moderate spinal stenosis without symptoms, previous lumbar spine surgeries (e.g., discectomies and laminectomies) if ​> ​6 months prior to baseline and no ongoing radicular symptoms, disc extrusions/protrusions ≤5 ​mm, and spondylolisthesis ≤2 ​mm. Compared to the RCT study, the CLBP prospective single-arm cohort study was more lenient in its enrollment criteria, allowing for inclusion of patients with extended-release opioid use and body mass index >40.

**Table 1:** Inclusion and Exclusion Criteria The primary inclusion and exclusion criteria for the two pooled studies are outlined below. Compared to the RCT study, the prospective single-arm cohort study allowed more lenient enrollment by eliminating the exclusion of patients using extended-release opioids and BMI >40.

Inclusion Criteria	Exclusion Criteria
• Skeletally mature patients with chronic (≥6 months) isolated lumbar back pain, who had not responded to at least 6 months of non-operative management • Type 1 or Type 2 Modic changes at one or more vertebral body for levels L3-S1 • Minimum ODI of 30 points (100-point scale) • Minimum VAS of 4 ​cm (10 ​cm scale) • Ability to provide informed consent, read and complete questionnaires	• MRI evidence of Modic changes at levels other than L3-S1 • Radicular pain (defined as nerve pain following a dermatomal distribution and that correlates with nerve compression in imaging) • Previous lumbar spine surgery (discectomy/laminectomy allowed if ​> ​6 months prior to baseline and radicular pain resolved) • Symptomatic spinal stenosis (defined as the presence of neurogenic claudication and confirmed by imaging) • Metabolic bone disease, spine fragility fracture history, trauma/compression fracture, or spinal cancer • Spine infection, active systemic infection, bleeding diathesis • Radiographic evidence of other pain etiology • Disc extrusion or protrusion >5 ​mm • Spondylolisthesis >2 ​mm at any level • Spondylolysis at any level • Facet arthrosis/effusion correlated with facet-mediated LBP • Beck Depression Inventory >24 or 3 or more Waddell’s signs • Compensated injury or litigation • Currently taking extended-release opioids with addiction behaviors^a^ • BMI >40^a^ • Bedbound or neurological condition that prevents early mobility or any medical condition that impairs follow-up^a^ • Contraindication to MRI, allergies to components of the device, active implantable devices, pregnant or lactating

Abbreviations: MRI - magnetic resonance imaging; ODI - Oswestry Disability Index; VAS - Visual Analogue Scale (average low back pain in past 7 days); mm - millimeters; BMI - body mass index.

a Exclusion criteria for the INTRACEPT trial only.

### 2.3 Enrollment and follow-up visits

A combined total of 113 study participants with primary vertebrogenic pain (confirmed by a combination of clinical inclusion criteria and presence of Modic Type 1 and/or Type 2 changes on MRI) were treated with BVNA in the treatment arm of the INTRACEPT RCT (N ​= ​66) and the prospective single-arm cohort study (N ​= ​47). The main study protocols required up to 2 years of follow-up (6 weeks and 3, 6, 9, 12 and 24 months) for the RCT and up to 12 months (6 weeks and 3, 6, 9 and 12 months) for the single-arm cohort study. MRIs were conducted at baseline and at 6-weeks post-BVNA. Baseline MRIs were reviewed by a single independent orthopedic spine medical reviewer to confirm Modic changes. Six-week MRIs were adjudicated by a single independent interventional radiologist for targeting success based on the degree of overlap of the ablation zone with the BVN and to confirm that all levels with Modic changes were treated. RCT study participants were approached at their last main study visit and the single-arm cohort study participants were approached at around 36 months post-BVNA to consent to participate in a long-term follow-up study at 3, 4, and 5 years.

### 2.4 Intervention

BVNA was conducted within each vertebral body with Modic changes (L3 to S1) using the same procedure technique at each investigative site with the Intracept ® System (Relievant Medsystems, Minneapolis, MN USA). The complete procedure and targeting success rates for each study were described previously [[13], [14], [15], [16], [17], [18], [19], [20]]. No targeting success requirement was applied for patient inclusion in this aggregate analysis, therefore all consenting patients who had the BVNA procedure are included.

### 2.5 Outcomes measures

Patient-reported clinical outcomes were collected at each study visit using validated questionnaires. The primary outcome in each original study was mean change in Oswestry Disability Index (ODI) from baseline to 3 months. Paired datasets (baseline and 3 years) for mean changes in ODI and numeric pain scores (NPS) were analyzed using a two-sided paired *t*-test with a 0.05 level of significance. The ODI questionnaire [21] was scored on a scale of 0 (no disability) to 100 (complete disability), with a minimal clinically important difference (MCID) of -15-points [22]. Low back pain was assessed using a subject-reported 10-point numeric pain scale (NPS) that is based on the Visual Analog Scale (VAS) pain rating questionnaire [23], where 0 represents no pain and 10 represents worst pain imaginable. Published MCID thresholds for pain improvement in CLBP are 1.5–2.0 points [22,23]. VAS scores were collected at baseline whereas NPS values were collected at 3-year telephonic follow-up visits. Patient-reported satisfaction, healthcare utilization (opioids, injections, additional pain interventions and surgeries), and pain impact on work/daily activity were also evaluated for the combined cohort.

### 2.6 Statistical analysis

Statistical analysis was performed with SAS version 9.4 software (SAS Institute Inc, Cary, NC). Aggregate and individual 3-year statistical analyses were performed for this reporting. Baseline characteristics are summarized using descriptive statistics. For categorical variables, the number (n) and percentages are reported. For continuous variables, the mean, standard deviation (SD), median, minimum, maximum, and confidence intervals are reported. Fisher’s exact tests were performed for each variable, using a 0.05 level of significance, to evaluate for poolability of the study populations.

Outcomes for pain and function were analyzed as observed (no imputations for missing data), last observation carried forward (LOCF), and intent-to-treat (ITT), with missing data treated as failure (zero reduction from baseline). Patient-reported ODI and NPS were compared at the 3-month primary endpoint between the full BVNA treatment cohort from the two studies and the cohort retained in the 3-year aggregate population, and also from baseline to 3 years in the 3-year aggregate population using a two-sided paired *t*-test with a 0.05 level of significance, as were difference. MCIDs of a 15-point improvement for ODI and a 50% improvement for NPS from baseline were used for treatment response thresholds in this study. Response rates at 3-years were analyzed using Fisher’s exact test with a 0.05 level of significance. Results for patient satisfaction, healthcare utilization and working status are summarized using descriptive statistics.

## 3 Results

### 3.1 Study participant disposition

**Fig. 1:**
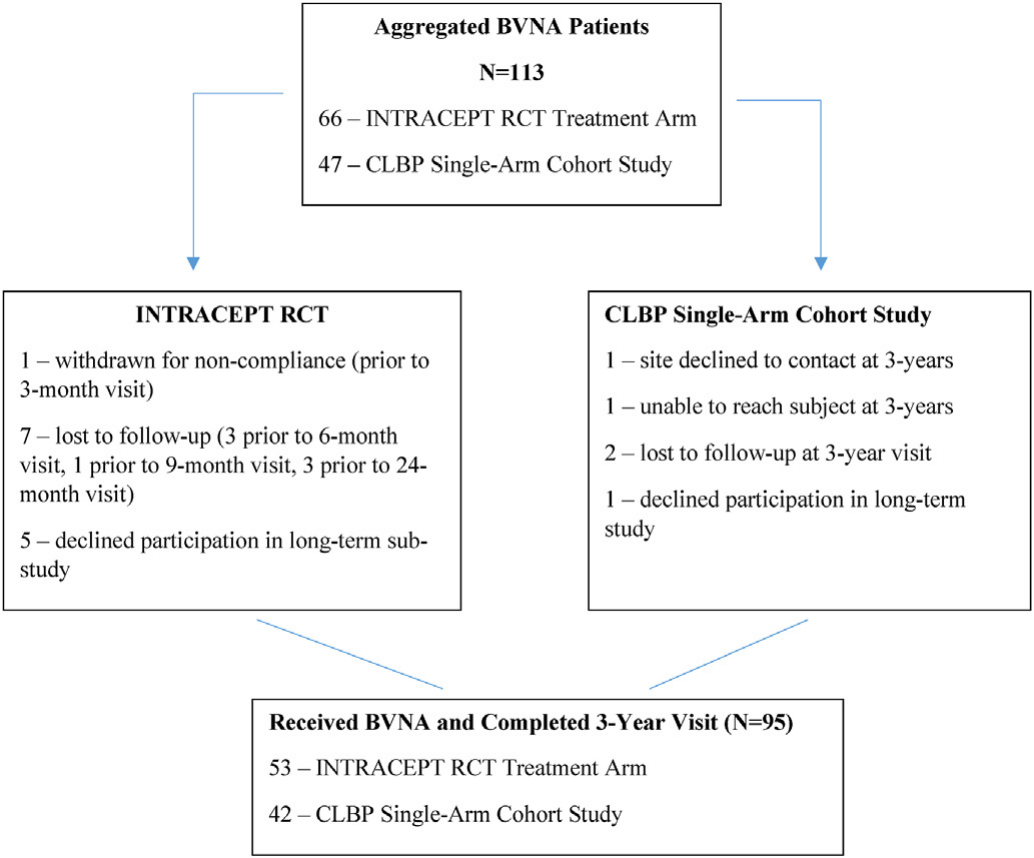
CONSORT Diagram, In the INTRACEPT RCT 140 participants were randomized when enrollment was stopped due to statistical superiority at the interim analysis, with sixty-six (66) randomized to BVNA. Fifty-three of these 66 consented to a long-term follow-up study (80% retention rate) and completed a 24-month and 3-year follow-up visits. In the prospective single arm cohort study, 47 participants were treated with BVNA. Of these, 42 consented to a long-term follow-up study (89% retention rate) and had a 3-year follow-up visit. Details on reasons for study exit prior to the 3-year visit are reported for this aggregate study within the CONSORT diagram below. Abbreviations: BVNA - basivertebral nerve ablation; CLBP - chronic low back pain; LTFU - lost to follow-up; RCT - randomized controlled trial.

This study population includes a total of 113 patients who underwent BVNA. Sixty-six (66) patients received BVNA in the treatment arm of the INTRACEPT RCT, which compared BVNA to standard care (66-BVNA, 74-standard care) through six months. Fifty-three (53) of the 66 in the randomized BVNA-treatment arm consented to participate in the long-term follow-up (participation rate of 80%) with all 53 completing a 3-year visit. In the original prospective single-arm cohort study, a total of 47 study participants received BVNA; 42 consented to participate in the long-term follow-up (participation rate of 89%) with all 42 completing a 3-year visit. The combined BVNA-treated participation rate at 3-year follow-up was 84% (95 of 113) for the two studies. Details on reasons for study exit are reported in Fig. 1.

### 3.2 Baseline clinical characteristics

Baseline characteristics for participants in the aggregate 3-year cohort are reported in Table 2 for the pooled and individual study results. The mean age was 48 years (30–68); 54% were female, 71% had back pain for ≥5 years, and 28% were actively taking opioids. One or more spinal injection treatment(s) had been performed within the 12 months preceding enrollment in 34% of participants, 13% had been treated with lumbar medial branch RFA, and 14% had prior low back surgery. Study participants reported severe pain and disability at baseline with a mean VAS of 6.7 ​± ​1.2 and mean ODI of 46.1 ​± ​10.8. There was a significant difference in participant age between the two study populations, and significantly fewer participants were taking opioids at baseline in the prospective single-arm cohort study. Given the clinical relevance and low level of significance, no adjustments were required for pooling the results.

**Table 2:** Three-Year Aggregate Cohort Baseline Clinical Characteristics Demographic features, low back pain treatment history, and clinical characteristics at baseline for BVNA study participants in the aggregate 3-year cohort are reported for the pooled and individual study results.

	INTRACEPT BVNA Treatment Arm (N ​= ​53)	CLBP Single Arm Cohort (N ​= ​42)	P-Value^a^	BVNA Aggregate Cohort (N ​= ​95)
**Mean Age in Years (range)**	50.6 (30–68)	45.4 (30–66)	0.0070	48.3 (30–68)
**Female, n (%)**	27 (50.9%)	24 (57.1%)	0.6790	51 (53.7%)
**Duration LBP ≥ 5 years, n (%)**	36 (67.9%)	31 (73.8%)	0.1170	67 (70.5%)
**Mean ODI (range)**	45.2 (30–76)	47.3 (30–72)	0.3430	46.1 (30–76)
**Mean VAS (range)**	6.6 (4–9)	6.8 (4–9)	0.2614	6.7 (4–9)
**Mean SF-36 (PCS) (range)**	32.2 (18–46)	32.4 (19–48)	0.8632	32.3 (18–48)
**Mean SF-36 (MCS) (range)**	54.1 (33–70)	53.7 (20–68)	0.8518	53.9 (20–70)
**Mean EQ-5D-5L (range)**	0.6 (0–1)	0.6 (0–1)	0.5458	0.6 (0–1)
**Mean Beck Depression Index (range)**	5.8 (0–16)	4.6 (0–13)	0.1653	5.3 (0–16)
**Opioid Use at Baseline, n (%)**	20 (37.7%)	7 (16.7%)	0.0382	27 (28.4%)
**Spinal Injection Treatment(s) (12 month prior to enrollment), n (%)**	20 (37.7%)	12 (28.6%)	0.3883	32 (33.7%)
**Pain Interventions (i.e., medial branch RFA), n (%)**	9 (17.0%)	3 (7.1%)	0.2166	12 (12.6%)
**Prior lumbar surgery (discectomy/laminectomy)**	7 (13.2%)	6 (14.3%)	1.0000	13 (13.7%)

^a^P-values using Fisher’s Exact test for categorical data and independent sample *t*-test for continuous data.

Abbreviations: BVNA - basivertebral nerve ablation; N - number; ODI - Oswestry disability index; VAS - visual analog scale; PCS - physical component score; MCS - mental component score.

### 3.3 Baseline imaging characteristics

Imaging data (endplate and motion segment descriptive characteristics) at baseline were read by a single independent radiologic reviewer and are provided in the supplementary materials (Tables S1 and S2). To report results at a participant level, the treated level (endplate and adjacent motion segment) with the greatest bone marrow intensity change (BMIC) height was evaluated for imaging characteristics. In the pooled cohort (N ​= ​95), 57.8% had Type 1 Modic changes and 42.2% had Type 2 Modic changes. Nearly 28% of participants in the pooled cohort were found to have increased facet joint fluid signal on MRI, 85% showed some degree of facet arthropathy (with 23% having moderate-to-large osteophytes), 20% had disc protrusion, and 12% had marked foraminal stenosis.

### 3.4 Aggregate cohort - basivertebral nerve ablation (BVNA) treatment

All vertebral levels with Modic changes present were treated in the two individual studies. A blinded independent interventional radiologist confirmed targeting as well as treatment of all Modic-involved levels. Vertebral bodies that were treated in each study and the 3-year aggregate cohort are reported in the supplementary materials (Table S3). The most common vertebral levels for treatment in this cohort were L5 at 97.9%, followed by S1 at 65.3%, L4 at 51.6%, and L3 at 7.4%. There were no significant differences in vertebral levels treated between the two study populations included in the aggregate cohort.

### 3.5 Three-month endpoint comparison between main study and 3-year aggregate cohorts

To assess for potential bias in outcomes within the volunteers comprising the 3-year BVNA aggregate study, the 3-month primary endpoints for pain and functional improvement (ODI, VAS/NPS, and response rates) were compared between the full BVNA treatment cohort (N ​= ​113) from the two studies and those comprising the 3-year BVNA aggregate population (N ​= ​95). No statistically significant differences were found for change in ODI, VAS, or responder rates for the 3-month primary endpoint between main study participants who declined the long-term sub-study and those who participated.

### 3.6 Aggregate cohort 3-year pain (NPS) and function (ODI) results

In the aggregate cohort of BVNA-treated patients with a 3-year visit, statistically significant improvements in pain (NPS) and function (ODI) were observed when compared to baseline. Mean changes in ODI and NPS were assessed for the following: as observed (no imputations for missing data), last observation carried forward (LOCF), and intent-to-treat (ITT) with missing data considered failure (improvement of zero from baseline values). In the as observed analysis, BVNA-treated participants reported a mean improvement in ODI of -31.2 ​± ​13.62 (p<0.0001; 95% CI 28.4 to 34.0) from a baseline score of 46.1 to 14.9 ​at 3 years post-BVNA. Mean improvement in NPS from baseline VAS was -4.3 ​± ​2.29 (p<0.0001; 95% CI 3.8 to 4.8) from a baseline score of 6.7 to 2.4 ​at 3 years post-BVNA. These mean improvements were similar in the LOCF and ITT analyses (see Table 3, Table 4).

**Table 3:** Aggregate Cohort ODI from Baseline to 3 Years Paired t-tests demonstrated significant improvements (p<0.0001) from baseline to 3 years in ODI for all three analyses conducted: 1) as observed, 2) last observation carried forward (LOCF), and 3) intent-to-treat (where missing data was deemed a failure with a zero reduction from baseline).

Oswestry Disability Index (ODI)	Aggregate Cohort BVNA As Observed (N ​= ​95)	Aggregate Cohort BVNA Last Observation Carried Forward (N ​= ​113^b^)	Aggregate Cohort BVNA ITT (Missing Data ​= ​Fail) (N ​= ​113)
**Baseline ODI**			
Mean+/-SD (range)	46.1 ​± ​10.79 (30 to 76)	45.8 ​± ​10.77 (30 to 76)	45.8 ​± ​10.77 (30 to 76)
[95% CI]	[43.9, 48.3]	[43.8, 47.8]	[43.8, 47.8]
**3-Year ODI**			
Mean+/-SD (range)	14.9 ​± ​13.28 (0 to 52)	15.6 ​± ​14.50 (0 to 62)	19.6 ​± ​16.73 (0 to 70)
[95% CI]	[12.2, 17.7]	[12.9, 18.3]	[16.5, 22.7]
**Improvement in ODI**			
Mean+/-SD (range)	−31.2 ​± ​13.62 (-10 to 70)	−30.0 ​± ​15.84 (-12 to 70)	−26.2 ​± ​16.95 (-10 to 70)
[95% CI]	[28.4, 34.0]	[27.1, 33.0]	[23.1, 29.4]
**P-Value**^**a**^	<0.0001	<0.0001	<0.0001

^a^P-values were calculated using a two-sided paired *t*-test.

^b^N ​= ​113 ​at baseline; n ​= ​111 with at least one ODI collected post BVNA; baseline value carried forward to 3-years in patients without any ODI measurements post BVNA.

Abbreviations: BVNA - basivertebral nerve ablation; N - number; ODI - Oswestry disability index; SD ​- ​standard deviation; CI - confidence interval.

**Table 4:** Aggregate Cohort Numeric Pain Score (NPS) from Baseline to 3 Years Paired t-tests demonstrated significant improvements (p<0.0001) from baseline to 3 years for VAS/NPS for all three analyses conducted: 1) as observed, 2) last observation carried forward (LOCF), and 3) intent-to-treat (where missing data was deemed failure with a zero reduction from baseline).

Numeric Pain Score (NPS)	Aggregate Cohort BVNA As Observed (N ​= ​95)	Aggregate Cohort BVNA Last Observation Carried Forward (N ​= ​113^b^)	Aggregate Cohort BVNA ITT (Miss ​= ​Fail) (N ​= ​113)
**Baseline VAS**			
Mean ​± ​SD (range)	6.7 ​± ​1.16 (4 to 9)	6.8 ​± ​1.2 (4 to 10)	6.8 ​± ​1.2 (4 to 10)
[95% CI]	[6.5, 6.9]	[6.5, 7.0]	[6.5, 7.0]
**3-Year NPS**			
Mean ​± ​SD (range)	2.4 ​± ​2.15 (0 to 8)	2.8 ​± ​2.57 (0 to 10)	5.2 ​± ​2.55 (0 to 10)
[95% CI]	[2.0, 2.8]	[2.3, 3.3]	[4.7, 5.7]
**Improvement in NPS**			
Mean ​± ​SD (range)	−4.3 ​± ​2.29 (-1 to 9)	−4.0 ​± ​2.56 (-3 to 9)	−1.6 ​± ​2.45 (-1 to 8)
[95% CI]	[3.8, 4.8]	[3.5, 4.5]	[1.1, 2.0]
**P-Value**^**a**^	<0.0001	<0.0001	<0.0001

^a^P-value calculated using two-sided paired *t*-test.

^b^N ​= ​113 ​at baseline, n ​= ​112 with at least one VAS/NPS collected post BVNA; baseline value carried forward to 3-years in patients without any VAS/NPS measurements post BVNA.

Abbreviations: BVNA - basivertebral nerve ablation; N - number; VAS - visual analog scale; SD ​- ​standard deviation; CI - confidence interval; NPS - numeric pain score.

### 3.7 Aggregate cohort 3-year pain (NPS) and function (ODI) categorical outcomes

**Fig. 2:**
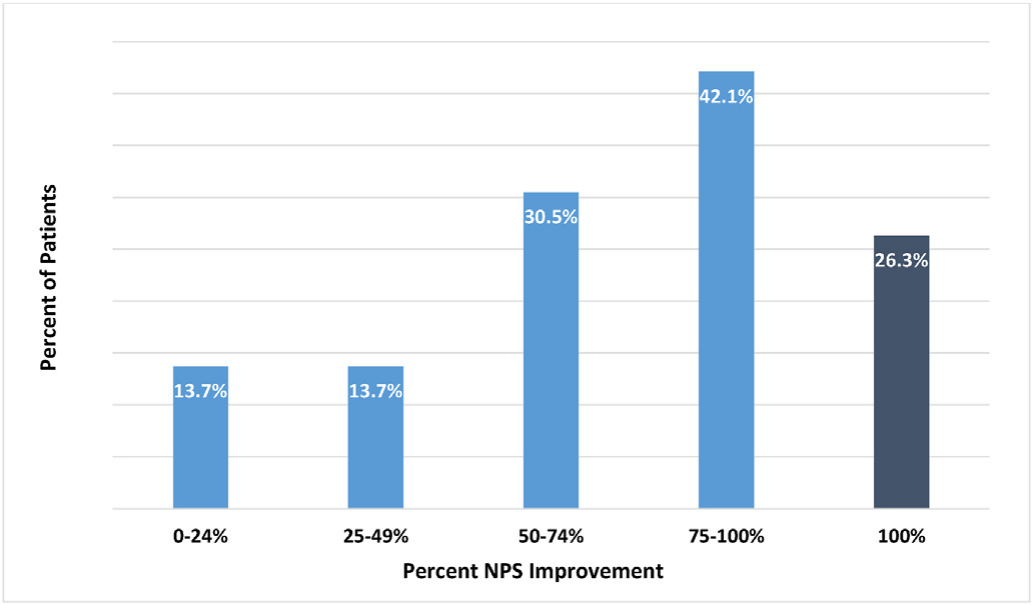
Percent of Patients by % NPS Improvement at 3 ​Years Post-BVNA, This figure depicts the proportion of study participants (N ​= ​95) by their percentage of NPS reduction from baseline. Seventy-three percent reported a ≥50% reduction in NPS and 26.3% of patients were pain-free with 100% pain reduction at 3 years post-BVNA. Abbreviations: NPS - numeric pain score; N – number.

At 3 years post-BVNA, 73% of aggregate cohort participants reported a ≥50% reduction in NPS, 42.1% experienced a ≥75% reduction, and 26.3% of participants were pain-free with 100% NPS reduction from baseline (see Fig. 2). Using a MCID of ≥50% improvement in pain, the proportion of responders was significantly greater than the proportion of non-responders (p<0.0001)

### 3.8 Aggregate cohort 3-year responder rates

Response rates using the pre-specified MCID thresholds for changes compared to baseline were as follows: ≥15-point improvement in ODI, ≥50% reduction in NPS, or ≥2 point reduction in NPS [22,23]. These were evaluated as observed, LOCF, and ITT (missing data recognized as no change from baseline and failed response) at the 3-year endpoint. Eighty-five percent (85%) of participants in the as observed analysis reported a ≥15-point ODI reduction (p<0.0001), 72.6% reported a NPS improvement of ≥50% (p<0.0001), and the combined responder rate (≥15-point improvement in ODI and ≥50% reduction in NPS from baseline) was 69.5% (p ​= ​0.0001). Response rates were similar between the as observed and the LOCF analysis. All responder rates were statistically significant except the ITT combined responder rate of ODI ≥15-point AND NPS ≥50% reduction which failed to reach significance at p ​= ​0.0739 (see Table 5).

**Table 5:** Three-Year Aggregate Cohort Responder Rates The proportion of responders was significant for function (ODI ≥15-point reduction) and pain (NPS ≥50% reduction) for all three analyses (as observed, LOCF, and ITT). Likewise, combined response rates were significant for ≥15-point AND NPS ≥2.0-point improvements for all three analyses. However, combined response rates of ODI ≥15-point AND NPS ≥50% reduction were significant for as observed and LOCF analyses only.

Responder Rates	BVNA As Observed (N ​= ​95)	BVNA LOCF (N ​= ​113^b^)	BVNA ITT (Miss ​= ​Fail) (N ​= ​113)
ODI ≥15-point reduction	81/95 (85.3%)	89/111 (80.2%)	81/113 (71.7%)
P-value^a^	<0.0001	<0.0001	<0.0001
NPS ≥50% reduction	69/95 (72.6%)	79/112 (70.5%)	69/113 (61.1%)
P-value^a^	<0.0001	<0.0001	0.0187
ODI ≥15-point AND NPS ≥50% reduction	66/95 (69.5%)	73/111 (65.8%)	66/113 (58.4%)
P-value^a^	0.0001	0.0009	0.0739
ODI ≥15-point AND NPS ≥2.0-point reduction	76/95 (80.0%)	83/111 (74.8%)	76/113 (67.3%)
P-value^a^	<0.0001	<0.0001	0.0002

^a^ P-values from chi-square tests.

^b^N ​= ​113 ​at baseline; n ​= ​111 with at least one ODI collected post BVNA; baseline value carried forward to 3-years in patients without any ODI measurements post-BVNA.

Abbreviations: BVNA - basivertebral nerve ablation; N - number; ODI - Oswestry disability index; NPS - numeric pain score.

### 3.9 Aggregate cohort 3-year patient satisfaction

At 3 years eighty-four percent (84%) of study participants reported improvement of their condition post-BVNA with 58% indicating “vastly improved.” Eleven percent (11%) reported no change in their condition and 3% reported their condition had worsened. Seventy-one percent (71%) of participants reported resuming the activity level they had enjoyed prior to onset of their low back pain, and 86% indicated they would have the procedure again for the same condition.

### 3.10 Aggregate cohort 3-year LBP impact on work

Of study participants who were working at baseline, twenty-three percent (23%) reported missing work for an average of 1.9 days due to LBP in the two weeks prior to baseline. Three patients were unable to work due to LBP at baseline. At 3 years, only 4.3% of working patients reported missing work for an average of 1.0 days due to LBP in the prior two weeks (a reduction of 83.3%) and only one patient reported being unable to work due to LBP. At baseline, 21 patients (22%) averaged 2.7 days in the past two weeks where they spent more than half the day in bed; this was reduced by 71% to 6 patients at 3 years post BVNA, with an average of 3.2 days in the past two weeks where they spent more than half the day in bed.

### 3.11 Aggregate cohort 3-year healthcare utilization

Twenty-eight percent (28%) of patients in the 3-year aggregate cohort were taking opioids at the time of study enrollment compared to 7.4% (7/95) who reported active opioid use within 30 days of the 3-year visit, representing a 74% reduction. Thirty-four percent (32/95) of patients in the aggregate cohort had one or more therapeutic spinal injections in the 12 months preceding baseline. During the 3 years post-BVNA only five (5.3%) patients had therapeutic spinal injection(s) that were adjudicated to be related to the same vertebral level and pain etiology per independent physician review, representing an 84% reduction from baseline. Seven (7.4%) patients reported another radiofrequency intervention or surgical intervention during the 3 years post-BVNA. Five (5.3%) of these patients were adjudicated by an independent clinical event committee as treatment for the same pain source and vertebral levels (2-lumbar fusion, 1-total disc replacement, 4-medial branch/facet joint RFA, 1-reintervention with BVNA). Three patients (3.2%) had 6 radiofrequency interventions or surgeries that were adjudicated as treatment for a different pain etiology and/or vertebral levels (1-lumbar fusion, 1-foraminotomy, 1-lateral branch/SI joint RFA, 3-medial branch/facet joint RFA).

### 3.12 Aggregate cohort 3-year adverse events

No serious device or device-procedure related adverse events were reported through 3 years. Seventeen participants in the full cohort of N ​= ​113 reported non-serious device-procedure related events post-BVNA (16 leg pain events and 1 inability to complete the procedure due to hardened bone). The majority of events were reported early in the follow-up period with no further events reported after 12 months. Post-procedure leg pain events were primarily treated with oral medication with a median resolution time of 56 days.

## 4 Discussion

This report provides pooled 3-year results for BVNA-treated participants from two clinical trials. With these results, the current study provides additional information regarding the long-term effectiveness and durability of pain and functional improvements in patients treated with BVNA for primary vertebrogenic CLBP. We report statistically significant and clinically meaningful improvements in paired analyses from baseline through 3 years post-ablation in this aggregate analysis. We also report individual study results which demonstrated significant improvements for both NPS and ODI for each study timepoint through 3 years. In comparing the three analyses (as observed, LOCF, and ITT), all three demonstrated significant improvements from baseline, including when missing data was conservatively deemed a treatment failure (zero improvement from baseline). The retention rate for long-term evaluation was good (84%) with no statistical differences between this study group and those lost to follow-up in their outcomes at the 3-month primary endpoints. Indeed, the LOCF analysis demonstrates that 50% of the participants who either declined the long-term follow-up or were lost to follow-up prior to 3 years reported ≥50% NPS reduction, and 38% reported they had achieved 100% pain relief in their last visit prior to study exit.

Results in this study are consistent with previously published long-term results of BVNA treatment from the pivotal RCT [15] which also reported a mean NPS reductions of 4.4 points at 5 years. Likewise, a mean ODI improvement of 31.2 points demonstrated in this study are similar to the mean ODI improvement of 25.95 points at 5 years in the pivotal RCT treatment arm [15]. While more participants in the aggregate analysis reported ≥50% reduction in NPS (73% compared to 66% in the pivotal RCT treatment arm at 5 years), the rate of participants reporting complete pain-relief at 5 years is higher (34%) compared to that at 3 years (26%). This pattern of prolonged benefit with gradual further improvement over time is not unexpected as statistically significant improvements in both NPS and ODI were maintained in the long-term follow-up of the pivotal RCT BVNA treatment arm at 24 months and 5 years, along with additional incremental improvements observed between these two timepoints [15]. This demonstrates the durability of improvements in pain and function offered by BVNA treatment and the subsequent favorable natural history. Healthcare utilization was significantly reduced from baseline during the 3 years following BVNA in this aggregate population with a 74% reduction in active opioid use and an 84% reduction in steroid injections for the same vertebral level and pain etiology. These rates are consistent with published 5-year results where 73% of patients stopped opioid usage and 93% fewer patients received one or more steroid injection post-BVNA [15]. Rates of low back surgeries and pain interventions were also low post-BVNA in this aggregate analysis with only 5 participants (5.3%) having another RF ablation procedure or surgery that was adjudicated by an independent clinical event committee to be treatment for the same pain source and vertebral levels. Of these, one participant (1%) had lumbar segmental fusion, 2 (2%) had medial branch RFA, 1 (1%) had a total disc replacement, and 1 (1%) had a repeat BVNA (without improvement). The low rate of spine interventions and surgeries following BVNA is notable in a population with greater than 5 years of CLBP producing severe self-reported pain and functional limitation, where 14% had prior lumbar surgeries and an independent MRI review revealed that the majority had severe disc degeneration (Pfirrmann Grade 4 in 44%, and Grade 5 in 36%) with many also having other common degenerative findings (28% had increased facet fluid on MRI, and 6% had olisthesis). Indeed, these rates are low compared to the rates observed in studies of hundreds-of-thousands of U.S. patients using private insurance care claims data (MarketScan® Commercial Claims and Encounters Database) where the rate of medial branch RFA is 26.7% during the first year after a facet joint injection [24], and the rate of spinal surgery after epidural steroid injection is 16.9% at 1 year and 26.1% at 5 years [25]. Combined, these findings indicate a substantial reduction in healthcare utilization for CLBP at 3 years post-BVNA for properly identified patients with vertebrogenic pain.

All participants in this aggregate analysis of two clinical trials were diagnosed with primary vertebrogenic pain, yet it is important to emphasize that this is not a condition that is exclusive of other spinal pathologies. Within this cohort, 14% had prior low back surgeries, 21% had a disc protrusion, 12% had marked foraminal stenosis, 23% had narrowing of facet joint space(s) and/or moderate-to-large osteophytes, 28% had increased facet fluid, and 6% had olisthesis. Significant clinical improvements combined with the reduced utilization of opioids, spine interventions, and surgeries over the 3-year period in this study suggests that BVNA treatment is beneficial even in the presence of other radiographic spinal pathology.

We think the success of BVNA is largely attributable to a few key factors. Specifically, the pathophysiology of vertebral endplate pain was extensively studied, defined, and linked to an objective biomarker (Modic changes) allowing for the identification of a specific phenotype of CLBP patients. Also, a treatment was developed specifically for this phenotype, interrupting the transmission of pain from its source. Similar success has only been achieved in other CLPB phenotypes with reliable and valid diagnostic selection criteria, including the treatment of lumbar radicular pain from a radiographically confirmed and corresponding source of nerve impingement [26], and the treatment of lumbar zygapophyseal joint pain with medial branch RFA based on response to dual diagnostic medial branch blocks [27]. Specific treatment of specific sources of back pain should produce robust outcomes.

Differentiation of vertebrogenic CLBP from other potential sources is informed by clinical assessment including pain location (i.e. midline verses lateral pain), and movements that exacerbate pain (i.e. activity verses rest and flexion versus extension), in addition to imaging confirmation of Type 1 and/or Type 2 Modic changes. Vertebrogenic pain is identified as midline low back pain, typically without radiation, that is exacerbated by forward flexion and sitting [28]. Vertebral endplate pain has been correlated with Type 1 and Type 2 Modic changes. Recent evidence showed that the presence of Modic changes, when used as an objective biomarker of vertebrogenic pain, was the only predictor of response to BVNA [29]. Pain and functional improvements were similar for Type 1 and Type 2 Modic changes and for the amount of Modic change based on height and overall area; for example, participants with Modic changes localized to the endplate responded similarly to those with Modic changes involving >50% of the vertebral height [30].

Modic changes are thought to be a late indicator of vertebral endplate damage [4], with systematic reviews reporting that erosive vertebral endplate defects are strongly associated with low back pain (odds ratio of 2.69) [31]. Our study supports Modic changes as an accurate biomarker even in the setting of other segmental findings: 80% of participants had DDD Pfirrmann grade 4/5 (grading scale 1 to 5), 49% had ≥50% disc height loss, and 16% had annular high intensity zones. Clinical studies on earlier biomarkers using advanced imaging and serum for identification of vertebrogenic pain are promising and continued research will hopefully benefit future patients [[32], [33], [34], [35]]. One example is single-photon emission computed tomography (SPECT-CT) that has been presented as a potential earlier biomarker of vertebral endplate damage. One concern of using SPECT-CT to identify vertebrogenic pain is the inability of SPECT-CT to detect Modic Type 2 changes that entail fatty marrow deposits [36,37]. Given that Modic Type 2 is more prevalent than Modic Type 1 [38], a substantial number of patients that may benefit from BVNA could be missed. Still, use of Modic changes has proven a successful imaging biomarker for the identification of vertebrogenic pain resulting in BVNA treatment response rates of 64% (95% CI 43–82%) for ≥50% VAS reductions and 75% (95% CI 63–85%) for a ≥15-point improvement in ODI at 12 months following BVNA in a recent meta-analysis of 414 patients treated with BVNA in six independent and sponsored trials [39].

Strengths of this aggregate analysis are that the two included cohorts were homogenous using similar inclusion/exclusion criteria, and that the study timeframes and study endpoints were collected uniformly. In addition, the attrition at 3 years was low with an 84% retention rate. Another strength is the similarity of these outcomes to those observed in a separate long-term study following BVNA [15]. Limitations of this study are the open-label design, industry sponsorship, and the lack of a long-term comparator group within the two studies, though the average improvement without BVNA would be expected to follow outcomes reported from non-surgical care where ODI improvement was only 7.4 points [40].

## 5 Conclusions

These results support vertebrogenic pain as a distinct and identifiable source of anterior column CLBP with BVNA producing statistically significant, clinically meaningful, and durable improvements in pain and function through 3 years in patients with primary vertebrogenic low back pain. BVNA-treated patients significantly reduced opioid use and interventions for low back pain.

## Declaration of competing interest

The authors declare the following financial interests/personal relationships which may be considered as potential competing interests:Kevin Macadaeg reports financial support was provided by Relievant Medsystems Inc. Kevin Macadaeg reports a relationship with Relievant Medsystems Inc that includes: consulting or advisory, funding grants, speaking and lecture fees, and travel reimbursement.
