# Supplementary material for: Intraosseous basivertebral nerve ablation: Pooled long-term outcomes from two prospective clinical trials
Source: Interv Pain Med. 2023 Jun 10;2(2):100256. doi: 10.1016/j.inpm.2023.100256 (PMC11373002; doi:10.1016/j.inpm.2023.100256)
Supplement: Multimedia component 1 [file mmc1.docx]

**Table S1 - Three-Year Aggregate Cohort Baseline MRI Endplate Characteristics**

Endplate characteristics from independent radiologic review of the baseline MRI for BVNA-treated study participants in the aggregate 3-year cohort are reported for pooled and individual study results. To report data at a participant level, the endplate with the greatest bone marrow intensity change (BMIC) height was selected alone for this comparison.

| **Baseline characteristics** | **INTRACEPT**  **BVNA Treatment Arm  (N=53)** | **CLBP**  **Single**  **Arm Cohort**  **(N=42)** | **Fisher's Exact**  **P-value^a^** | **BVNA Aggregated Cohort (N=95)** |
| --- | --- | --- | --- | --- |
| **BMIC Type** |  |  | 0.520255 |  |
| **1 - Type 1** | 54.0% (27/50) | 62.5% (25/40) | . | 57.8% (52/90) |
| **2 - Type 2** | 46.0% (23/50) | 37.5% (15/40) | . | 42.2% (38/90) |
| **BMIC Height** |  |  | 0.602694 |  |
| **1 - localized to endplate only** | 26.0% (13/50) | 35.0% (14/40) | . | 30.0% (27/90) |
| **2 - less than 25% of vertebral body height** | 40.0% (20/50) | 37.5% (15/40) | . | 38.9% (35/90) |
| **3 - 25 to 50% of vertebral body height** | 32.0% (16/50) | 22.5% (9/40) | . | 27.8% (25/90) |
| **4 - more than 50% vertebral body height** | 2.0% (1/50) | 5.0% (2/40) | . | 3.3% (3/90) |
| **BMIC Area** |  |  | 0.269478 |  |
| **1 - less than 25% of endplate area** | 20.0% (10/50) | 35.0% (14/40) | . | 26.7% (24/90) |
| **2 - 25 to 50% of endplate area** | 34.0% (17/50) | 30.0% (12/40) | . | 32.2% (29/90) |
| **3 - more than 50% of endplate area** | 46.0% (23/50) | 35.0% (14/40) | . | 41.1% (37/90) |
| **Endplate Defect** |  |  | 0.050234 |  |
| **0 - No** | 10.0% (5/50) | 27.5% (11/40) | . | 17.8% (16/90) |
| **1 - Yes** | 90.0% (45/50) | 72.5% (29/40) | . | 82.2% (74/90) |
| **Endplate Defect Shape** |  |  | 0.489739 |  |
| **1 - sharp, angular** | 2.2% (1/45) | 3.4% (1/29) | . | 2.7% (2/74) |
| **2 - Schmorl’s node** | 6.7% (3/45) | 0 | . | 4.1% (3/74) |
| **3 - irregular** | 91.1% (41/45) | 96.6% (28/29) | . | 93.2% (69/74) |
| **Endplate Defect Size** |  |  | 0.023000 |  |
| **1 - less than 1/3 endplate area** | 15.6% (7/45) | 31.0% (9/29) | . | 21.6% (16/74) |
| **2 - between 1/3 and 2/3 endplate area** | 28.9% (13/45) | 44.8% (13/29) | . | 35.1% (26/74) |
| **3 - more than 2/3 endplate area** | 55.6% (25/45) | 24.1% (7/29) | . | 43.2% (32/74) |

^a^P-value using Fisher’s Exact test for comparison of individual study populations

Abbreviations: BVNA - basivertebral nerve ablation; N - number; BMIC - bone marrow intensity changes
