# Supplementary material for: Intraosseous basivertebral nerve ablation: Pooled long-term outcomes from two prospective clinical trials
Source: Interv Pain Med. 2023 Jun 10;2(2):100256. doi: 10.1016/j.inpm.2023.100256 (PMC11373002; doi:10.1016/j.inpm.2023.100256)
Supplement: Multimedia component 2 [file mmc2.docx]

**Table S2 - Three-Year Aggregate Cohort Baseline MRI Motion Segment Characteristics**

Motion segment characteristics from independent radiologic review of the baseline MRI for BVNA-treated participants in the aggregate 3-year cohort are reported for the pooled and individual study results. To report data at a participant level, the motion segment that adjoins the treated endplate with the greatest BMIC height was included in this report.

| **Baseline characteristics** | **INTRACEPT BVNA Treatment Arm (N=53)** | **CLBP Single**  **Arm Cohort**  **(N=42)** | **Fisher's Exact P-value^a^** | **BVNA Aggregated Cohort (N=95)** |
| --- | --- | --- | --- | --- |
| **Degenerative Disc Disease** |  |  | 0.625363 |  |
| **0 - inhomogeneous structure with or without horizontal bands** | 0.0% (0/50) | 2.5% (1/40) | . | 1.1% (1/90) |
| **1 - inhomogeneous structure with gray disc** | 16.0% (8/50) | 22.5% (9/40) | . | 18.9% (17/90) |
| **2 - inhomogeneous structure with gray to black disc** | 46.0% (23/50) | 42.5% (17/40) | . | 44.4% (40/90) |
| **3 - inhomogeneous structure with black disc** | 38.0% (19/50) | 32.5% (13/40) | . | 35.6% (32/90) |
| **Nuclear Signal** |  |  | 0.636753 |  |
| **0 - normal, pure white signal on T2-weighted images** | 2.0% (1/50) | 2.5% (1/40) | . | 2.2% (2/90) |
| **1 - moderate loss, intermediate between normal and severe** | 14.0% (7/50) | 22.5% (9/40) | . | 17.8% (16/90) |
| **2 - severe loss, homogenous black signal** | 84.0% (42/50) | 75.0% (30/40) | . | 80.0% (72/90) |
| **Disc Height** |  |  | 0.197392 |  |
| **0 - normal, less than 10% loss of expected height** | 12.0% (6/50) | 15.0% (6/40) | . | 13.3% (12/90) |
| **1 - moderate narrowing, 10-50% loss** | 46.0% (23/50) | 27.5% (11/40) | . | 37.8% (34/90) |
| **2 - severe narrowing, 50% loss** | 42.0% (21/50) | 57.5% (23/40) | . | 48.9% (44/90) |
| **High Intensity Zone** |  |  | 0.772007 |  |
| **0 - No** | 86.0% (43/50) | 82.5% (33/40) | . | 84.4% (76/90) |
| **1 - Yes** | 14.0% (7/50) | 17.5% (7/40) | . | 15.6% (14/90) |
| **Disc Contour** |  |  | 0.652721 |  |
| **0 - normal, no extension beyond the interspace** | 2.0% (1/50) | 5.0% (2/40) | . | 3.3% (3/90) |
| **1 - bulge, circumferential, symmetrical disc extension** | 78.0% (39/50) | 72.5% (29/40) | . | 75.6% (68/90) |
| **2 - protrusion, focal or asymmetrical disc extension** | 20.0% (10/50) | 20.0% (8/40) | . | 20.0% (18/90) |
| **3 - extrusion. focal disc extension beyond the interspace** | 0.0% (0/50) | 2.5% (1/40) | . | 1.1% (1/90) |
| **Nerve Root Compromise** |  |  | 0.912962 |  |
| **0 - No nerve root contact** | 88.0% (44/50) | 92.5% (37/40) | . | 90.0% (81/90) |
| **1 - Nerve root contact without deviation** | 8.0% (4/50) | 5.0% (2/40) | . | 6.7% (6/90) |
| **2 - Nerve root deviation** | 2.0% (1/50) | 2.5% (1/40) | . | 2.2% (2/90) |
| **3 - Nerve root compression/deformation** | 2.0% (1/50) | 0.0% (0/40) | . | 1.1% (1/90) |
| **Facet Joint Arthropathy** |  |  | 0.093711 |  |
| **0 - normal facet joint space (2–4 mm width)** | 4.0% (2/50) | 17.5% (7/40) | . | 10.0% (9/90) |
| **1 - narrowing of the FJ space (<2 mm) and/or small osteophytes** | 66.0% (33/50) | 65.0% (26/40) | . | 65.6% (59/90) |
| **2 - narrowing of the FJ space and/or moderate osteophytes** | 26.0% (13/50) | 17.5% (7/40) | . | 22.2% (20/90) |
| **3 - narrowing of the FJ space and/or large osteophytes** | 4.0% (2/50) | 0.0% (0/40) | . | 2.2% (2/90) |
| **Facet Joint Fluid** |  |  | 0.061159 |  |
| **0 - No** | 64.0% (32/50) | 82.5% (33/40) | . | 72.2% (65/90) |
| **1 - Yes** | 36.0% (18/50) | 17.5% (7/40) | . | 27.8% (25/90) |
| **Olisthesis** |  |  | 0.063181 |  |
| **0 - No** | 90.0% (45/50) | 100.0% (40/40) | . | 94.4% (85/90) |
| **1 - Yes** | 10.0% (5/50) | 0.0% (0/40) | . | 5.6% (5/90) |
| **Congenital Stenosis** |  |  | 1.000000 |  |
| **0 - No** | 98.0% (49/50) | 100.0% (40/40) | . | 98.9% (89/90) |
| **1 - Yes** | 2.0% (1/50) | 0.0% (0/40) | . | 1.1% (1/90) |
| **Foraminal Stenosis** |  |  | 0.248268 |  |
| **0 - normal foramina with normal dorsolateral border** | 24.0% (12/50) | 40.0% (16/40) | . | 31.1% (28/90) |
| **1 - slight foraminal stenosis and deformity of the epidural fat** | 62.0% (31/50) | 50.0% (20/40) | . | 56.7% (51/90) |
| **2 - marked foraminal stenosis and deformity of the epidural fat** | 14.0% (7/50) | 10.0% (4/40) | . | 12.2% (11/90) |
| **Central Spinal Stenosis** |  |  | 0.250936 |  |
| **0 - no constriction of thecal sac** | 94.0% (47/50) | 100.0% (40/40) | . | 96.7% (87/90) |
| **1 - mild constriction of thecal sac with minimal loss of CSF** | 6.0% (3/50) | 0.0% (0/40) | . | 3.3% (3/90) |
| **Lateral Regions Spinal Stenosis** |  |  | 1.000000 |  |
| **0 - No nerve root contact** | 98.0% (49/50) | 97.5% (39/40) | . | 97.8% (88/90) |
| **1 - Nerve root contact without deviation** | 2.0% (1/50) | 2.5% (1/40) | . | 2.2% (2/90) |

^a^P-value using Fisher’s Exact test for comparison of individual study populations.

Abbreviations: BVNA - basivertebral nerve ablation; N - number
