# Supplementary material for: Intraosseous basivertebral nerve ablation: Pooled long-term outcomes from two prospective clinical trials
Source: Interv Pain Med. 2023 Jun 10;2(2):100256. doi: 10.1016/j.inpm.2023.100256 (PMC11373002; doi:10.1016/j.inpm.2023.100256)
Supplement: Multimedia component 3 [file mmc3.docx]

**Table S3 - Three-Year Aggregate Cohort Vertebral Bodies Treated**

Vertebral bodies that were treated in the 3-year aggregate cohort are reported. The most common vertebral treatment levels in this cohort were L5 and S1.

| **Vertebral Bodies Treated – “Yes”** | **INTRACEPT BVNA Treatment Arm (N=53)** | **CLBP Single-Arm Cohort (N=42)** | **Fisher's Exact P-value^a^** | **BVNA Aggregated Cohort (N=95)** |
| --- | --- | --- | --- | --- |
| **L3, n (%)** | 5 (9.4%) | 2 (4.8%) | 0.4585 | 7 (7.4%) |
| **L4, n (%)** | 30 (56.6%) | 19 (45.2%) | 0.3060 | 49 (51.6%) |
| **L5, n (%)** | 52 (98.1%) | 41 (97.6%) | 1.0000 | 93 (97.9%) |
| **S1, n (%)** | 33 (62.3%) | 29 (69.0%) | 0.5226 | 62 (65.3%) |

^a^P-value shown for comparison of individual study populations; not applicable to the aggregated cohort.

Abbreviations: BVNA - basivertebral nerve ablation; N - number; L - lumbar; S - sacral
